# Knockdown of long non-coding RNA TP73-AS1 inhibits cell proliferation and induces apoptosis in esophageal squamous cell carcinoma

**DOI:** 10.18632/oncotarget.6963

**Published:** 2016-01-21

**Authors:** Wenqiao Zang, Tao Wang, Yuanyuan Wang, Xiaonan Chen, Yuwen Du, Qianqian Sun, Min Li, Ziming Dong, Guoqiang Zhao

**Affiliations:** ^1^ College of Basic Medical Sciences, Zhengzhou University, Zhengzhou, China; ^2^ Department of Hemato-tumor, The First Affiliated Hospital of Henan University of TCM, Zhengzhou, China; ^3^ Collaborative Innovation Center of Cancer Chemoprevention, Henan, China

**Keywords:** LncRNA TP73-AS1, BDH2, biomarker, chemosensitivity, esophageal cancer

## Abstract

Recent studies have shown that long non-coding RNAs (lncRNAs) are involved in a variety of biological processes and diseases in humans, including cancer. Our study serves as the first comprehensive analysis of lncRNA TP73-AS1 in esophageal cancer. We utilized a lncRNA microarray to analyze the expression profile of lncRNAs in esophageal squamous cell carcinoma. Our results show that lncRNA TP73-AS1 and BDH2 levels are generally upregulated in esophageal cancer tissues and are strongly correlated with tumor location or TNM stage in clinical samples. LncRNA TP73-AS1 knockdown inhibited BDH2 expression in EC9706 and KYSE30 cells, whereas BDH2 knockdown repressed esophageal cancer cell proliferation and induced apoptosis via the caspase-3 dependent apoptotic pathway. Overexpression of BDH2 in lncRNA TP73-AS1 knockdown cells partially rescued cell proliferation rates and suppressed apoptosis. In mouse xenografts, tumor size was reduced in lncRNA TP73-ASI siRNA-transfected tumors, suggesting that downregulation of lncRNA TP73-AS1 attenuated EC proliferation *in vitro* and *in vivo*. In addition, BDH2 or lncRNA TP73-AS1 knockdown enhanced the chemosensitivity of esophageal cancer cells to 5-FU and cisplatin. Our results suggest that lncRNA TP73-AS1 may be a novel prognostic biomarker that could serve as a potential therapeutic target for the treatment of esophageal cancer.

## INTRODUCTION

Esophageal cancer (EC) is a leading cause of cancer-related death that is especially prevalent in China [1, 2]. Esophageal squamous cell carcinoma (ESCC) and esophageal adenocarcinoma (EA) are the two main types of esophageal carcinoma. However, in China, 90% of cases involve squamous cell carcinoma [2]. EC is difficult to cure unless it is diagnosed at a very early stage, before metastasis. The five-year survival rate of EC patients remains very poor despite rapid advances in surgical techniques and therapies [3, 4]. There is an urgent need to identify new molecular markers and independent risk factors that are associated with EC development and progression to improve diagnosis, prevention and treatment.

Long non-coding RNAs (lncRNAs) are non-coding RNAs of more than 200 nucleotides (nt) in length and are characterized by diverse and complex sequences and mechanisms of action [5]. Some studies using deep transcriptome sequencing and microarrays have shown that 70-90% of the human genome is estimated to be transcribed into non-protein-coding RNA [6]. Recent studies indicate that lncRNAs are involved in a variety of biological processes and diseases in humans [5–8]. Notably, some lncRNAs serve as pivotal regulators in the development and progression of EC [9–11]. However, the expression, function, and molecular mechanism of lncRNA, including lncRNA TP73-AS1, in EC remain unclear.

Ours is the first comprehensive analysis of lncRNA TP73-AS1 in EC. We utilized lncRNA microarrays to analyze the expression profile of lncRNAs in ESCC. We investigated the function of lncRNA TP73-AS1 in the proliferation and apoptosis of ESCC cell lines, and we evaluated the expression and relevance of lncRNA TP73-AS1 in clinical ESCC. The contribution of lncRNA TP73-AS1 to ESCC malignancy and the molecular mechanisms underlying its action were also investigated. Our findings demonstrate that lncRNA TP73-AS1 could be a potential prognostic marker and therapeutic target for the treatment of EC.

## RESULTS

### LncRNA TP73-AS1 is upregulated in ESCC

**Figure 1:**
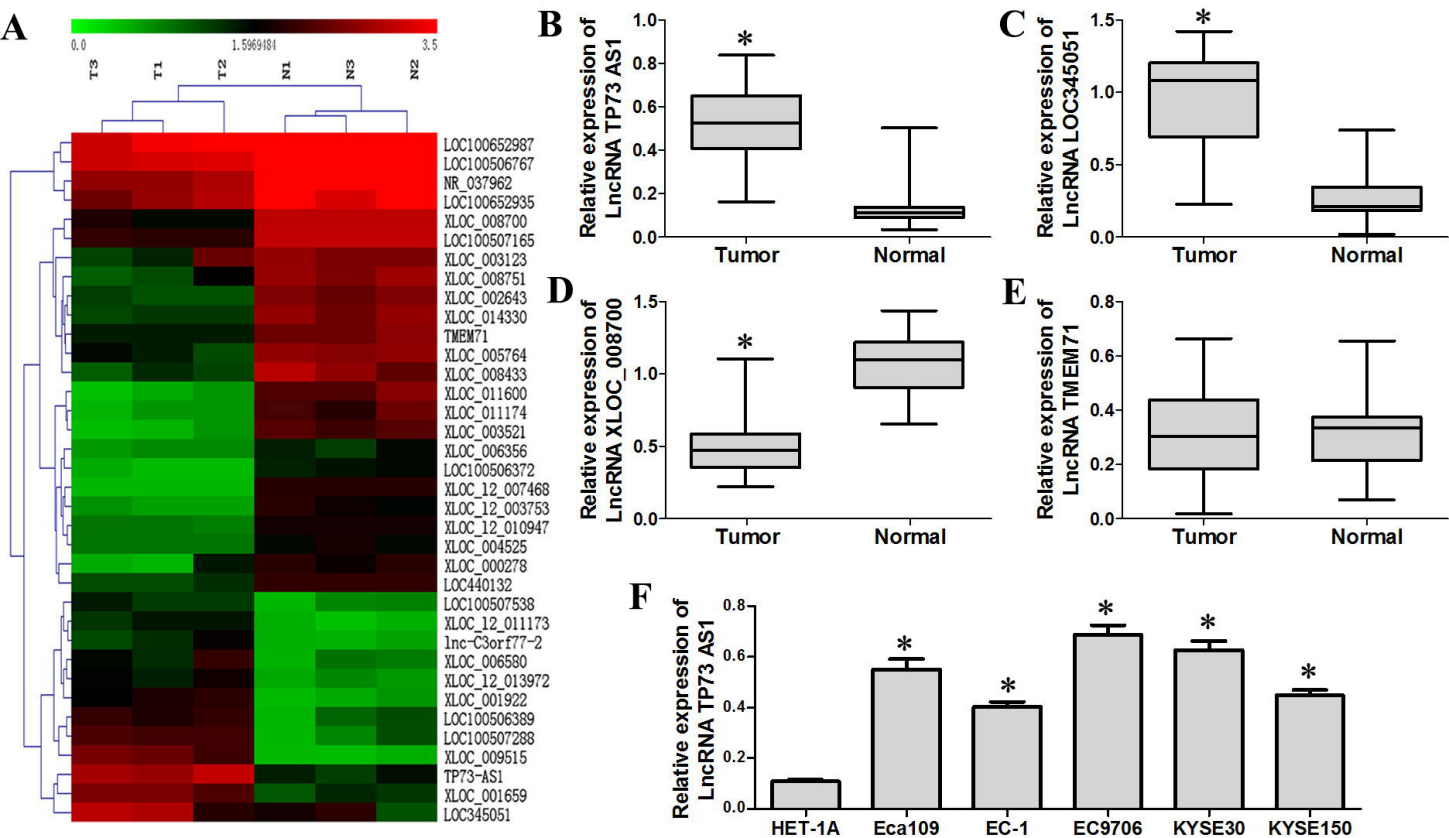
LncRNA TP73-AS1 is upregulated in EC **A.** LncRNA microarray analysis was performed on total RNA isolated from three esophageal squamous cell carcinoma tissue samples and corresponding adjacent normal esophageal tissues. T: tumor, N: normal. **B.** & **C.** qRT-PCR results show that LncRNA TP73-AS1 and LOC345051 levles in EC tissues was increased as compared to adjacent non-cancerous tissues. **D.** LncRNA XLOC_008700 expression in EC tissues was decreased as compared to adjacent non-cancerous tissues **E.** No significant difference was observed for lncRNA TMEM71 between EC tissues and corresponding adjacent normal esophageal tissues **F.** LnRNA TP73-AS1 expression was generally upregulated in EC cell lines (Eca109, EC-1, EC9706, KYSE30 and KYSE150) compared with the normal esophageal cell line, HET-1A. (*P < 0.05)

To identify unique genes involved in ESCC, lncRNA microarray analysis was performed using total RNA isolated from three ESCC tissue samples, and the corresponding adjacent normal esophageal tissues as the control. A fold change of >5.0 was set as the threshold for upregulated genes, and a fold change of <0.25 was set as the threshold for downregulated genes. The data were expressed as the mean ± SD. Relative to normal tissue, tumor tissue displayed differentially upregulated and downregulated lncRNAs (Figure 1A, Tables 1 & 2). We validated four lncRNAs microarray results using quantitative RT-PCR, including two upregulated lncRNAs (lncRNA TP73-AS1 and lncRNA LOC345051) and two downregulated lncRNAs (lncRNA XLOC_008700 and lncRNA TMEM71), in 60 pairs of primary ESCC tissues and their corresponding adjacent normal esophageal tissues. LncRNA TP73-AS1 and LOC345051 expression was significantly higher in ESCC tissues as compared to controls (*P*<0.05, Figure 1B & 1C), whereas that of lncRNA XLOC_008700 was significantly reduced (*P*<0.05, Figure 1D). No significant difference in the expression of lncRNA TMEM71 was observed (*P*>0.05, Figure 1E). A strong correlation between the results of quantitative RT-PCR and Agilent lncRNA microarray profiles was observed. Among the four lncRNAs examined, only the results for lncRNA TMEM71 did not match between the two methods (*P*>0.05).

**Table 1:** Important LncRNAs upregulated in esophageal cancer tissues

LncRNA Name (GeneSymbol)	Regulation	chr	Foldchange
LOC_009515	Up	chr11	56.88932665
LOC100508287	Up	chr19	50.65416524
XLOC_003940	Up	chr4	33.61186757
LOC100507288	Up	chr15	29.47021948
XLOC_l2_012175	Up	chr5	28.404181
XLOC_008441	Up	chr10	25.94510375
XLOC_001659	Up	chr2	20.90140762
XLOC_008604	Up	chr10	20.7259683
XLOC_l2_011327	Up	chr4	19.56286412
XLOC_001922	Up	chr2	18.0051049
LOC100506389	Up	chr6	17.1631327
TP73-AS1	Up	Chr1	15.43420213
XLOC_000984	Up	chr1	14.26167571
XLOC_003457	Up	chr4	11.30667087
LOC345051	Up	chr4	10.8862842
LOC100505535	Up	chr19	9.731238977
LOC100288728	Up	chr17	8.323208628
XLOC_l2_011173	Up	chr4	7.565944009
LOC100506791	Up	chr5	7.066572332
XLOC_000768	Up	chr1	6.62576346
XLOC_l2_013972	Up	chr7	5.982766676
XLOC_006580	Up	chr7	5.833858119
XLOC_013154	Up	chr19	5.829168883
XLOC_012095	Up	chr17	5.428283614
lnc-C3orf77-2	Up	chr3	5.420727934
LOC100507118	Up	chr15	5.176895871
XLOC_010348	Up	chr13	5.161127603
LOC100507538	Up	chr7	5.154945589
LOC100507546	Up	chr5	5.078538224

**Table 2:** Important LncRNAs downregulated in esophageal cancer tissues

LncRNA Name (GeneSymbol)	Regulation	chr	Foldchange
XLOC_003057	Down	chr3	0.249757395
LOC100652987	Down	chr14	0.246677494
XLOC_013794	Down	chr20	0.245043248
LOC100506965	Down	chr15	0.244963091
XLOC_006356	Down	chr7	0.243994579
LOC100506965	Down	chr15	0.243475481
XLOC_l2_010555	Down	chr4	0.240849695
XLOC_004525	Down	chr5	0.23899544
XLOC_l2_009303	Down	chr22	0.23479824
LOC100506948	Down	chr15	0.231482655
LOC100506767	Down	chr14	0.226170865
XLOC_010572	Down	chr13	0.214910906
NR_033708	Down	chr1	0.205847239
NR_037962	Down	chr1	0.196268697
LOC100506948	Down	chr15	0.193894798
XLOC_001508	Down	chr2	0.193804544
LOC440132	Down	chr13	0.170180059
XLOC_001699	Down	chr2	0.169704767
LOC100652867	Down	chr1	0.161978327
XLOC_l2_010947	Down	chr4	0.15706701
XLOC_005910	Down	chr6	0.154877739
LOC100506372	Down	chr16	0.136250705
TMEM71	Down	chr8	0.128252246
SDIM1	Down	chr6	0.1267782
LINC00403	Down	chr13	0.124328358
LOC100652935	Down	chr10	0.121165174
XLOC_005530	Down	chr6	0.118259755
XLOC_006734	Down	chr8	0.113602855
LOC100507165	Down	chr11	0.095550485
XLOC_007085	Down	chr8	0.090350118
LOC100507008	Down	chr10	0.078132901
XLOC_005764	Down	chr6	0.071876751
XLOC_000625	Down	chr1	0.067898838
XAGE-4	Down	chrX	0.065780332
XLOC_l2_003753	Down	chr13	0.065250732
XLOC_011470	Down	chr15	0.063513893
XLOC_l2_012023	Down	chr5	0.062435021
XLOC_003123	Down	chr3	0.062402559
LOC100507165	Down	chr11	0.051852056
XLOC_008390	Down	chr10	0.050919081
XLOC_l2_007468	Down	chr2	0.050499374
XLOC_011174	Down	chr15	0.049276821
XLOC_000278	Down	chr1	0.049040072
XLOC_014330	Down	chr22	0.04852727
XLOC_008700	Down	chr10	0.047007051
XLOC_002643	Down	chr3	0.04692143
XLOC_003786	Down	chr4	0.046616316
XLOC_008795	Down	chr10	0.046603541
XLOC_l2_000037	Down	chr1	0.046006394
XLOC_004598	Down	chr5	0.045762193
XLOC_008577	Down	chr10	0.045365941
XLOC_006870	Down	chr8	0.041676401
XLOC_000684	Down	chr1	0.041671585
XLOC_l2_005988	Down	chr17	0.038935609
XLOC_011603	Down	chr15	0.037427881
XLOC_005036	Down	chr5	0.036159627
XLOC_008751	Down	chr10	0.035382596
XLOC_008433	Down	chr10	0.03338041
XLOC_011600	Down	chr15	0.030349173
XLOC_003521	Down	chr4	0.025289366

We selected lncRNA TP73-AS1 located at 1p36.32 as our subsequent study candidate because of its high levels and similar expression patterns in different clinical specimens. We further examined lncRNA TP73-AS1 expression in the normal esophageal cell line, HET-1A, and EC cell lines Eca109, EC-1, EC9706, KYSE30 and KYSE150. LncRNA TP73-AS1 expression was generally upregulated in EC cell lines (Eca109, EC-1, EC9706, KYSE30, and KYSE150) (Figure 1F).

### LncRNA TP73-AS1 knockdown inhibits ESCC proliferation and induces apoptosis

**Figure 2:**
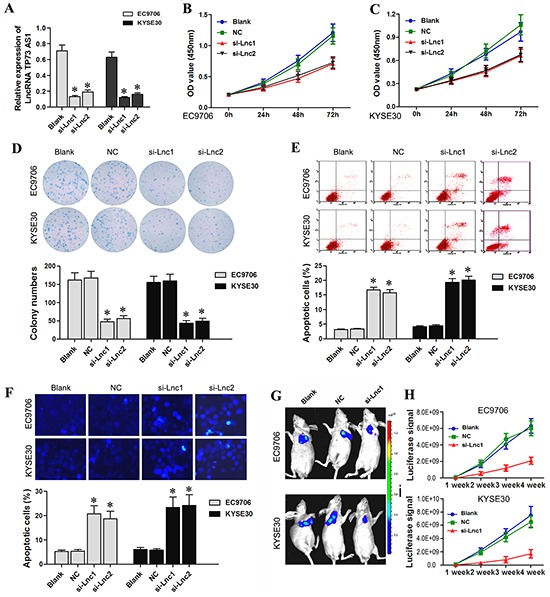
LncRNA TP73-AS1 siRNA inhibits EC cell proliferation and induces apoptosis **A.** qRT-PCR results showed that LncRNA TP73-AS1 expression was downregulated in EC9706 and KYSE30 cells transfected with lncRNA TP73-AS1 siRNA1 or siRNA2. **B.** As measured by CCK-8 assay, statistically significant decrease in EC9706 cell proliferation in the si-lnc1 and si-lnc2 groups was observed compared to the blank and NC groups **C.** A statistically significant decrease in KYSE30 cell proliferation in the si-lnc1 and si-lnc2 groups was observed. **D.** LncRNA TP73-AS1 siRNA1 or siRNA2 transfection of EC9706 and KYSE30 cells reduced colony formation. **E.** LncRNA TP73-AS1 siRNA induces EC9706 and KYSE30 apoptosis as assessed by flow cytometry. **F.** Hoechst 33342 staining showed that lncRNA TP73-AS1 siRNA1 or siRNA2 transfection led to a significant increase in EC9706 and KYSE30 cell apoptosis. **G.** LncRNATP73-AS1 siRNA attenuates the proliferation of EC cells *in vivo*. Tumor size was significantly lower in si-Lnc1 nude mice as compared to control mice **H.** Luciferase signal was significantly lower in si-Lnc1 nude mice as compared to control mice. Blank: un-transfected cells. NC: cells were transfected with nonsense siRNA. si-Lnc1: cells were transfected with lncRNATP73-AS1 siRNA1. si-Lnc2: cells were transfected with lncRNATP73-AS1 siRNA2. *P<0.05 compared to the control group.

Higher lncRNA TP73-AS1 levels in both EC tissues and cell lines as compared to non-cancer controls suggested that lncRNA TP73-AS1 might play an important role in EC tumorigenesis. We designed lncRNATP73-AS1 siRNA1 and siRNA2, which we transfected into EC9706 and KYSE30 cells using a recombinant lentivirus. qRT-PCR analysis showed that transfection of lncRNATP73-AS1 siRNA1 or siRNA2 into EC9706 and KYSE30 cells reduced lncRNA TP73-AS1 levels (Figure 2A). CCK-8 and colony formation assay were utilized to evaluate cell proliferation. The results showed that 24, 48 and 72 hours after transfection, EC9706 (Figure 2B) and KYSE30 cells (Figure 2C) that received lncRNATP73-AS1 siRNA1 or siRNA2 had reduced proliferation rates as compared to un-transfected (Blank) and nonsense siRNA transfected (NC) cells. LncRNATP73-AS1 siRNA also clearly suppressed colony formation (Figure 2D).

We also examined apoptosis with flow cytometry using the FITC Annexin V Apoptosis Detection Kit I (BestBio, Shanghai, China). The results showed that the number of early and late apoptotic cells at 48–96 hours post-siRNA-transfection was significantly higher in EC9706 and KYSE30 cells (Figure 2E) compared to controls. LncRNA TP73-AS1 knockdown also clearly induced apoptosis in EC9706 and KYSE30 cells as indicated by the Hoechst 33342 staining assay (Figure 2F).

To confirm the growth inhibitory effect of lncRNATP73-AS1 siRNA on EC *in vivo*, a xenograft tumor growth assay was performed. Tumor size and luciferase signal were significantly reduced in the lncRNATP73-AS1 siRNA mice group (EC9706 and KYSE30 cells transfected with lncRNATP73-AS1 siRNA1) as compared to control mice (NC and Blank groups) at the fourth week (*P*<0.05, Figure 2G & 2H). Our results showed that downregulation of lncRNA TP73-AS1 attenuated EC cell proliferation both *in vitro* and *in vivo*.

### LncRNA TP73-AS1 knockdown inhibits BDH2 expression in EC9706 and KYSE30 cells

**Figure 3:**
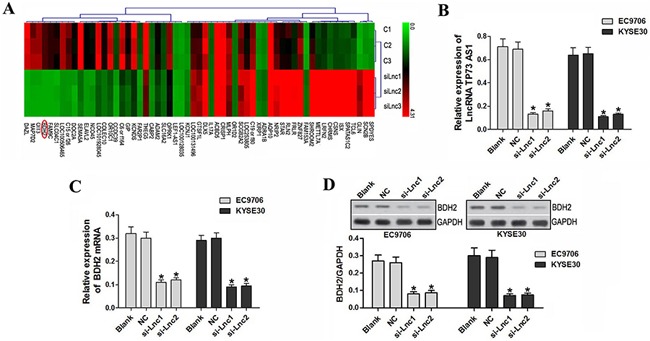
LncRNA TP73-AS1 knockdown inhibited BDH2 expression **A.** mRNA microarray analysis was performed using EC9706 and KYSE30 cells transfected with lncRNATP73-AS1 siRNA and nonsense siRNA. C1: EC9706 transfected with nonsense siRNA; C2: EC9706 transfected with nonsense siRNA; C3: KYSE30 transfected with nonsense siRNA; siLnc1: EC9706 transfected with lncRNATP73-AS1 siRNA1; siLnc2: EC9706 transfected with lncRNATP73-AS1 siRNA2; siLnc3: KYSE30 transfected with lncRNATP73-AS1 siRNA1. **B.** qRT-PCR results show that lncRNA TP73-AS1 expression was downregulated in EC9706 and KYSE30 cells transfected with lncRNATP73-AS1 siRNA1 or siRNA2. **C.** BDH2 expression was also decreased in lncRNATP73-AS1 siRNA1 and siRNA2 transfected cells. **D.** Western blot analysis showed that BDH2 protein was reduced in lncRNATP73-AS1 siRNA1 or siRNA2-transfected cells. (*P<0.05). Blank: un-transfected cells. NC: cells were transfected with nonsense siRNA. si-Lnc1: cells were transfected with lncRNATP73-AS1 siRNA1. si-Lnc2: cells were transfected with lncRNATP73-AS1 siRNA2. *P<0.05 compared to the control group.

To explore the molecular mechanisms of lncRNA TP73-AS1 in tumorigenesis, mRNA microarray analysis was performed using EC9706 and KYSE30 cells transfected with lncRNATP73-AS1 siRNA or nonsense siRNA. BDH2 expression was significantly decreased in EC9706 and KYSE30 cells transfected with lncRNATP73-AS1 siRNA compared to the control (*P*<0.05; Figure 3A).

BDH2 is a novel cytosolic-type 2-hydroxybutyrate dehydrogenase that plays a pivotal role in the utilization of cytosolic ketone bodies in the mitochondria, as well as the tricarboxylic acid cycle [12]. Guo, et al. identified BDH2 as a short-chain dehydrogenase/reductase family member, originally named DHRS6 [12]. Yang, et al. reported that BDH2 overexpression reduces overall survival and decreases patient response to intensive induction chemotherapy. Furthermore, the mechanism by which BDH2 works as an anti-apoptotic factor is mediated by survivin via the caspase-3 independent pathway [13].

To verify the effect of lncRNATP73-AS1 siRNA on BDH2, lncRNA TP73-AS1 (Figure 3B) and BDH2 mRNA levels were measured in EC9706 and KYSE30 cells by qRT-PCR. BDH2 expression was reduced in lncRNA TP73-AS1 siRNA1- or siRNA2-transfected cells (*P*<0.05; Figure 3C). Western blot analysis showed that the BDH2 protein levels were also significantly reduced (*P*<0.05; Figure 3D) in siRNA-transfected cells.

### BDH2 knockdown inhibits EC cell proliferation and induces apoptosis

**Figure 4:**
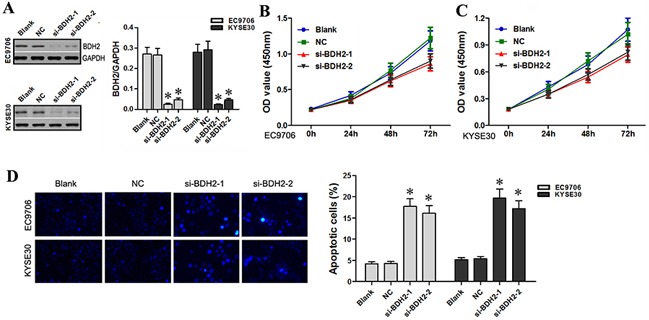
BDH2 knockdown inhibits EC cell proliferation and induces apoptosis **A.** Western blot and qRT-PCR analyses showed that protein and mRNA levels of BDH2 were lower in BDH2 siRNA1 or siRNA2 transfected EC9706 and KYSE30 cells. **B.** & **C.** As measured by CCK-8 assay, decreased EC9706 and KYSE30 cell proliferation was observed in BDH2 siRNA1 or siRNA2 transfected cells. **D.** Hoechst 33342 staining assay showed that BDH2 siRNA induced apoptosis in EC9706 and KYSE30 cells (*P<0.05). Blank: un-transfected cells. NC: cells were transfected with nonsense siRNA. si-BDH2-1: cells were transfected with BDH2 siRNA1. si-BDH2-2: cells were transfected with BDH2 siRNA2. *P<0.05 compared to the control group.

BDH2 protein and mRNA levels were significantly decreased in BDH2 siRNA1- or siRNA2-transfected EC9706 and KYSE30 cells as compared to controls (Figure 4A). CCK-8 assay results showed that at 24, 48 and 72 h hours post-transfection, BDH2 siRNA-transfected cells exhibited significantly reduced proliferation (Figure 4B & 4C). These results suggested that BDH2 might function as a tumor suppressor in EC cells *in vitro*.

Next, we evaluated apoptosis using Hoechst 33342 staining. The number of apoptotic cells post-BDH2 siRNA transfection significantly increased in EC9706 and KYSE30 cells as compared to controls (Figure 4D). These results suggest that BDH2 knockdown inhibits EC cell proliferation and induces apoptosis, which is similar to the effects of lncRNA TP73-AS1 siRNA on EC cells. The results of our study are in agreement with the findings of Yang, et al. that BDH2 works as an anti-apoptotic factor [13].

### BDH2 or lncRNA TP73-AS1 knockdown activates apoptosis protein expression and enhances chemosensitivity to 5-FU and cisplatin

**Figure 5:**
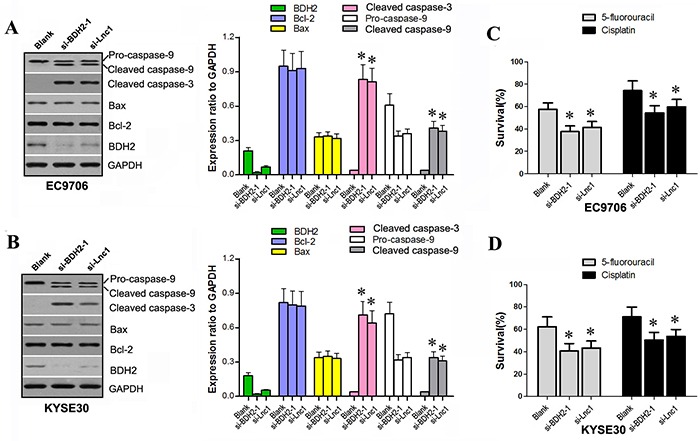
BDH2 or lncRNA TP73-AS1 knockdown activates apoptosis protein expression and enhances chemo-sensitivity to 5-FU or cisplatin in EC cells **A.** & **B.** Western blot analysis showed that BDH2 or lncRNATP73-AS1 knockdown enhanced expression of cleaved caspase-3 and cleaved caspase-9 in EC9706 and KYSE30 cells. **C.** & **D.** Knockdown of BDH2 or lncRNA TP73-AS1 also enhanced chemo-sensitivity to 5-FU or cisplatin in EC9706 and KYSE30 cells. (*P<0.05). Blank: un-transfected cells. si-BDH2-1: cells were transfected with BDH2 siRNA1. si-Lnc1: cells were transfected with lncRNATP73-AS1 siRNA1.

We have shown that both BDH2 siRNA and lncRNATP73-AS1 siRNA induce apoptosis. To explore the molecular mechanisms involved, we transfected EC9706 and KYSE30 cells with lncRNATP73-AS1 siRNA1 or BDH2 siRNA1. Western blot was used to confirm expression of related proteins. BDH2 expression significantly decreased in EC9706 and KYSE30 cells after BDH2 siRNA1 or lncRNATP73-AS1 siRNA1 transfection (Figure 5A & 5B). Levels of cleaved caspase-3 and cleaved caspase-9 proteins were enhanced in BDH2 siRNA1 or lncRNATP73-AS1 siRNA1-transfected cells. However, levels of Bcl-2, Bax and pro-caspase-9 were not significantly different following transfection (Figure 5A & 5B).

Previous studies have described two distinct but convergent pathways that initiate apoptotic responses: the death receptors and mitochondrial pathways [14–16]. The mitochondrial apoptosis pathway is regulated by caspase-9 [15–19]. Initiators of caspase-9 ultimately lead to increased mitochondrial permeability, thereby facilitating the release of cytochrome c (Cyt-c) from the inter-mitochondrial membrane space into the cytosol [20–22]. Cyt-c in turn activates caspase-9 through the apoptosome, triggering the activation of caspase-3, thereby resulting in apoptosis. Therefore, both activated caspase-8 (death receptor pathway) and caspase-9 (mitochondrial pathway) mobilize caspase-3 (the key executioner caspase), which in turn causes cellular apoptosis [23–25]. Our results suggest that BDH2 siRNA and lncRNATP73-AS1 siRNA induce apoptosis via a caspase-3 dependent pathway. Apoptosis likely contributes to inhibition of growth in BDH2 or lncRNA TP73-AS1 knockdown EC cells.

To determine the effect of BDH2 siRNA and lncRNATP73-AS1 siRNA on ESCC cells sensitivity to the chemotherapeutics, 5-FU and cisplatin, cell survival rates were measured. Transfection of EC9706 and KYSE30 cells with BDH2 siRNA1 or lncRNATP73-AS1 siRNA1 resulted in decreased cell survive rates following treatment with 5-FU or cisplatin (*P*<0.05; Figure 5C & 5D). Therefore, knockdown of BDH2 or lncRNATP73-AS1 enhanced the chemosensitivity of EC cells to 5-FU and cisplatin.

### BDH2 overexpression partially rescues proliferation rates and suppresses apoptosis in lncRNATP73-AS1 downregulated cells

**Figure 6:**
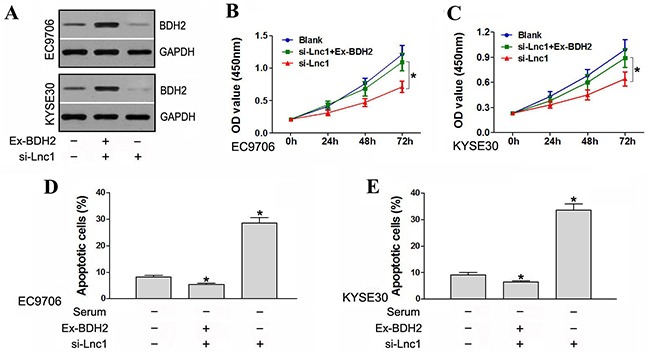
BDH2 overexpression partially rescued cell proliferation and suppressed apoptosis in lncRNA TP73-AS1 downregulated cells **A.** Transfection with pcDNA3.1-BDH2 increased BDH2 expression. Ex-BDH2: pcDNA3.1-BDH2. si-Lnc1: lncRNATP73-AS1 siRNA1. **B.** & **C.** BDH2 overexpression partially rescued proliferation rates in lncRNATP73-AS1 downregulated EC9706 and KYSE30 cells. Blank: un-transfected cells. si-Lnc1: cells were transfected with lncRNATP73-AS1 siRNA1. si-Lnc1+Ex-BDH2: cells were transfected with lncRNATP73-AS1 siRNA1 and pcDNA3.1-BDH2. **D.** & **E.** BDH2 overexpression suppresed apoptosis in lncRNA TP73-AS1 downregulated EC9706 and KYSE30 cells. (*P<0.05).

We constructed the expression vector, pcDNA3.1-BDH2, which we then transfected into EC9706 and KYSE30 cells. Western blot analysis showed that transfection increased BDH2 expression (Figure 6A).

A CCK-8 assay was conducted to evaluate cell proliferative capacity. LncRNATP73-AS1 siRNA1-transfected cells again exhibited a reduced proliferation rate as compared to controls. However, when lncRNATP73-AS1 downregulated cells were co-transfected with pcDNA3.1-BDH2, overexpression of BDH2 was found to partly rescue proliferation rates (Figure 6B & 6C). Apoptosis assays indicated that lncRNA TP73-AS1 knockdown increased apoptosis induced by serum starvation in EC9706 and KYSE30 cells. However, co-transfection with pcDNA3.1-BDH2 suppressed apoptosis in lncRNA TP73-AS1 downregulated cells (Figure 6D & 6E). Thus, BDH2 overexpression partially rescued proliferation rates and suppressed apoptosis in lncRNA TP73-AS1 downregulated cells.

### LncRNATP73-AS1 and BDH2 expression are related to EC TNM stage

**Figure 7:**
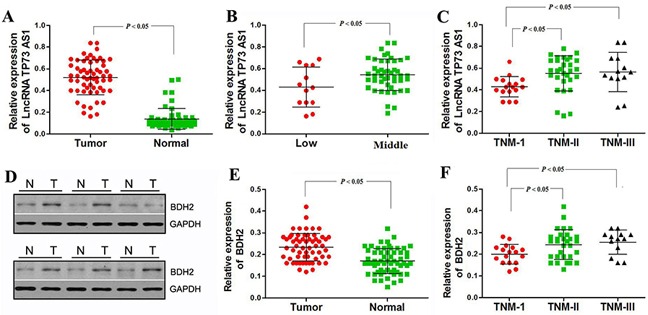
LncRNA TP73-AS1 and BDH2 expression correlate with TNM stage in EC **A.** As shown by real-time PCR analysis, relative expression of lncRNA TP73-AS1 was significantly upregulated in EC tissues. **B.** Relative expression of lncRNA TP73-AS1 was downregulated in low tumor locations. **C.** LncRNA TP73-AS1 expression was lower in TNM stage I tumors. **D.** Western blot analysis showed that BDH2 protein was significantly upregulated in EC tissues. **E.** Real-time PCR analysis showed that BDH2 mRNA was upregulated in EC tissues. **F.** BDH2 expression was lower in TNM stage I tumors.

To study BDH2 and lncRNA TP73-AS1 levels in EC tissues, western blot and real-time PCR analyses were conducted in 60 primary EC tissues and 60 normal esophageal tissues adjacent to tumors. The relationship between lncRNATP73-AS1 and BDH2 levels and clinicopathologic characteristics in EC patients is summarized in Table 3. LncRNA TP73-AS1 and BDH2 levels were significantly upregulated in EC tissues (Figure 7A, 7D & 7E).

**Table 3:** Expression of LncRNA and BDH2 in ESCC cases

Parameter	n	LncRNA		BDH2	
		Expression level	*P* value	Expression level	*P* value
Gender					
Male	39	0.53±0.179	0.611	0.23±0.068	0.390
Female	21	0.51±0.118		0.24±0.053	
Age(years)					
<60	21	0.49±0.171	0.297	0.23±0.066	0.941
≥60	39	0.54±0.153		0.23±0.63	
Location					
Low	13	0.43±0.183	0.022*	0.23±0.066	0.609
Middle	47	0.54±0.145		0.24±0.062	
Differentiation					
Well	17	0.50±0.181	0.760	0.24±0.071	0.799
Moderate	32	0.52±0.135		0.23±0.060	
Poor	11	0.55±0.199		0.24±0.064	
TNM stage					
I	17	0.43±0.095	0.017*	0.20±0.045	0.024*
II	30	0.52±0.163		0.24±0.069	
III	13	0.57±0.181		0.26±0.061	
Lymph node metastasis					
Negative	40	0.51±0.161	0.430	0.23±0.063	0.977
Positive	20	0.54±0.158		0.23±0.065	

* Indicated statistical significance (P<0.05)

LncRNA TP73-AS1 expression was also strongly correlated with tumor location and TNM stage, but not with gender, age, differentiation or lymph metastasis (Table 3). BDH2 expression was strongly correlated to TNM stage only. LncRNA TP73-AS1 expression was lower in low EC locations and during stage I, whereas middle EC locations and stages II–III exhibited higher levels, which were indicative of a significant correlation between lncRNA TP73-AS1 level and clinicopathologic stage (Figure 7B & 7C, Table 3). Similarly, BDH2 expression was lower during stage I, whereas stages II–III showed higher levels, indicating a correlation between BDH2 expression and TNM stage (Figure 7F, Table 3). These results provide strong evidence that lncRNA TP73-AS1 and BDH2 expression are closely related to the TNM stage of EC.

## DISCUSSION

In recent years, there has been rapid progress in both the experimental technologies and computational prediction algorithms for lncRNA discovery. So far 58,648 lncRNA genes have been identified [26, 27]. Current studies have shown that lncRNAs play key roles in diverse biological processes such as embryonic development, cell growth and tumorigenesis by regulating gene expression at the transcriptional and post-transcriptional levels [28–34]. LncRNA TP73-AS1 is located on human chromosomal band 1p36.32. TP73-AS1 is the antisense of the coding gene TP73, which encodes a product that shares structural and functional characteristics with TP53 [35]. Global genomic analysis has shown that up to 70% of protein-coding transcripts have antisense partners, and the perturbation of the antisense RNA alters the expression of the sense gene [36]. TP73-AS1 covers substantial portions of TP73, suggesting that TP73-AS1 may function by posttranscriptional regulation of TP73 gene expression [37]. To the best of our knowledge, information on lncRNA TP73-AS1 in ESCC is still limited.

Our study serves as the first comprehensive analysis of lncRNA TP73-AS1 in EC. We selected lncRNA TP73-AS1 as the target gene based on lncRNA profiles, and then designed lncRNA TP73-AS1 siRNA to block TP73-AS1 expression, and thus attenuate proliferation and invasion of ESCC. We found that lncRNA TP73-AS1 siRNA inhibits EC cell proliferation and induces cell apoptosis by regulating BDH2. Our results also suggest that downregulation of lncRNA TP73-AS1 and BDH2 induced apoptosis via a caspase-3 dependent pathway. These results are in agreement with the findings of Yang, et al. that BDH2 works as an anti-apoptotic factor [13]. It is also possible that there are other factors that could contribute to the regulation of this protein. Yu, et al. reported that lnRNA TP73-AS1 is significantly downregulated in non-small cell lung cancer as compared to normal lung tissues (*P*<0.001), but it is strongly upregulated in large-cell carcinoma specimens compared to adenocarcinoma, small-cell lung cancer and squamous cell carcinoma tissues [37]. These findings indicate that lnRNA TP73-AS1 may play an important role in the development and progression of various tumors.

Our results demonstrated that lncRNA TP73-AS1 could be a novel prognostic biomarker and a potential target for the treatment of EC. In ESCC, altered expression of lncRNAs has previously been reported. Guifeng Wei, et al. identified the potential tumor suppressor lncRNA Epist in ESCC, and provided a basis for future efforts to identify functional lncRNAs for therapeutic targeting [38]. Zhang X, et al. provided the first evidence that there is correlation between CCAT2 expression and poor survival in ESCC [39]. These studies indicate that deregulation of lncRNAs may promote ESCC carcinogenesis.

Caspase activation plays a central role in cell apoptosis. Caspase-3 and caspase-9 are the most frequently activated cell death proteases, and catalyze the specific cleavage of various key cellular proteins [24]. Our western blot assay showed that cleaved caspase-3 and cleaved caspase-9 protein levels were enhanced in BDH2 and lncRNA TP73-AS1 knockdown cell lines, respectively. However, expression of Bcl-2, Bax and pro-caspase-9 did not significantly differ following siRNA transfection. We inferred that knockdown of lncRNA TP73-AS1 or BDH2 induced cell apoptosis via a caspase-3 dependent apoptosis pathway.

Previous reports have described response rates ranging from 25% to 60% for chemotherapy in the treatment of solid tumors, in particular those of the head and neck [40–43]. Preoperative chemotherapy using cisplatin or 5-FU in combination with radiation therapy has also been used for the treatment of ESCC. Cisplatin and 5-FU are thought to induce radiosensitization in these tumors [37]. We found that BDH2 or lncRNA TP73-AS1 knockdown enhanced chemosensitivity to 5-FU or cisplatin in EC cells.

In summary, our study serves as a first comprehensive analysis of lncRNA TP73-AS1 function in EC, and provides strong evidence that lncRNA TP73-AS1 may be a novel prognostic biomarker and a potential therapeutic target for the treatment of EC.

## MATERIALS AND METHODS

### Patients and tissue samples

Sixty primary esophageal squamous cell cancer tissue samples and the adjacent normal esophageal tissues were collected. All samples were obtained from patients who were treated at the First Affiliated Hospital of Zhengzhou University and the Henan Tumor Hospital of Zhengzhou University in Zhengzhou, China between 2010 and 2013. The age distribution of patients was 63.2 ± 7.2 years. After the frozen specimens were stained with hematoxylin and eosin and examined by surgical pathologists, they were then snap frozen in liquid nitrogen. Normal esophageal tissues adjacent to the tumors were used as controls. None of the cancer patients in the present study had received preoperative radiation or chemotherapy. Informed consent was obtained from each of the patients. The Research Ethics Committee of Zhengzhou University approved the present study.

### LncRNA expression microarray analysis

Three pairs of primary ESCC tissues and corresponding adjacent normal esophageal tissues were analyzed by lncRNA microarray analysis (SurePrint Human Gene Expression Microarray Kit, Agilent Technologies, Santa Clara, CA, USA). Microarray analysis was performed using 5 μg of total RNA extracted from histologically confirmed cancer and corresponding adjacent normal tissues. The BROAD Institute database was used in the development of the array. After hybridization and washing, processed slides were scanned with an Agilent Microarray Scanner (Agilent Technologies, Santa Clara, CA, USA). Raw data were extracted as pair files using the Feature Extraction software 10.7 (Agilent Technologies). The data were expressed as the mean ± SD. Raw data were normalized using a Quantile algorithm provided by Gene Spring Software 11.0 (Agilent Technologies).

### Cell culture

The human esophageal cell line, HET-1A, and EC cell lines Eca109, EC-1, EC9706, KYSE30 and KYSE150 were purchased from the Type Culture Collection of the Chinese Academy of Sciences (Shanghai, China). The cell lines were cultured in RPMI-1640 medium supplemented with 10% fetal bovine serum (FBS, Gibco, Australia), 100 U/mL penicillin, and 50 μg/mL streptomycin, and were kept in incubators with humidified atmospheres of 5% CO_2_ and 95% air at 37°C.

### Lentiviral constructs and transfections

Recombinant lentiviral vectors carrying lncRNA TP73-AS1 siRNA1, TP73-AS1 siRNA2, BDH2 siRNA1 or BDH2 siRNA2 were constructed with standard molecular techniques. EC9706 and KYSE30 cells were infected with the recombinant lentivirus to generate stably transfected cells. Concentrated lentiviruses were transfected at a multiplicity of infection (MOI) of 40 in RPMI-1640 without FBS. The supernatant was replaced with complete culture medium after 24 hours. The expression of lncRNA TP73-AS1 and BDH2 in infected cells was validated by quantitative real-time polymerase chain reaction (qRT-PCR). LncRNA TP73-AS1 and BDH2 siRNA sequences are in Supplementary Table S1.

### qRT-PCR

Total RNA was extracted from EC tissue samples and adjacent non-tumor tissue samples using the TRIzol reagent (Invitrogen, Carlsbad, CA, USA), according to the manufacturer's instructions. Approximately 1 μg of RNA was used to synthesize cDNA. The expression levels of BDH2 and lncRNA TP73-AS1 were determined by qPCR (ABI 7500fast system, Applied Biosystems, CA, USA), using GAPDH as the endogenous control. qRT-PCR results were expressed relative to the ratio of lncRNA TP73-AS1 or BDH2 and GAPDH expression. The forward and reverse primer sequences for LnRNATP73-AS1 are 5′ CCGGTTTTCCAGTTCT TGCAC 3′ and 5′GCCTCACAGGGAAACTTCATGC3′, respectively. For BDH2: forward 5′ TTCCAGCGTCAAAGGAGTTGT 3′, reverse 5′ TTCCTGGGCA CACACAGTTG 3′.

### Western blot analysis

Total protein was extracted from the cells using RIPA buffer containing phenylmethanesulfonylfluoride (PMSF). Total protein concentrations were measured by using a BCA Protein Assay Kit (Pierce, Rockford, IL). Thirty μg of the protein lysate was subjected to sodium dodecyl sulfate-polyacrylamide gel electrophoresis (SDS-PAGE) and transferred onto PVDF membranes. The PVDF membranes were blocked with 5% BSA in 0.05% Tween 20-TBS for 1 hour and incubated with the corresponding primary antibody diluted in blocking buffer overnight at 4°C. Dilutions for primary antibodies were as follows: anti-BDH2 (1:1,000, Santa Cruz Biotech, Dallas, TX, USA), anti-BCL-2 (1:400, Santa Cruz Biotech), anti-BAX (1:1,000, Santa Cruz Biotech), anti-cleaved-caspase-9 (1:1,000, Santa Cruz Biotech), anti-pro-caspase-9 (1:1000, Santa Cruz Biotech), and anti-cleaved caspase-3 (1:1,000, Santa Cruz Biotech). After extensive washing with TBST, anti-rabbit IgG-HRP secondary antibody (1:5,000, Santa Cruz Biotech) was added. Signals were determined using a chemiluminescence detection kit (Amersham Pharmacia Biotech, Piscataway, NJ, USA).

### Cell proliferation assay

Esophageal cancer cells were transferred into 96-wells plates at a density of 1×10^4^ cells/well, with five replicate wells per group. The relative number of viable cells was detected using Cell Counting Kit-8 reagents (CCK-8; Dojindo, Japan) at 0, 24, 48 and 72 hours. Results were recorded using a microplate reader (Elx800; BioTek, VT, USA) at a wavelength of 450 nm. Experiments were conducted in triplicate.

### Colony formation assay

ESCC cells were seeded and transfected with lncRNA TP73-AS1. Cells were suspended in RPMI-1640 containing 0.35% low-melting agarose and plated onto 0.6% agarose in six-well culture plates at a density of 1×10^5^ cells per dish. The plates were incubated for two weeks at 37°C in a 5% CO_2_ incubator, and the number of colonies was counted after staining with 0.1% crystal violet solution. Colonies with more than 50 cells were manually counted. Experiments were done in triplicate.

### Flow cytometry

Cell apoptosis was detected by flow cytometry. Human EC cells were harvested at 48 hours post-transfection by trypsinization. Tumor cells were resuspended in binding buffer at a density of 1×10^6^ cells/mL. After double-staining with FITC-Annexin V and propidium iodide (PI) using the FITC Annexin V Apoptosis Detection Kit I (BestBio, Shanghai, China), the cells were analyzed using a FACScan® flow cytometer equipped with Cell Quest software (BD Biosciences, San Jose, CA, USA) according to manufacturer's instructions. Experiments were performed in triplicate.

### Hoechst 33342 staining

EC9706 and KYSE30 cells were treated with different concentrations of a1-PDX for 48 hours, washed twice with cold PBS, and then incubated in the dark in 5 μL of Hoechst/PI staining buffer for 15 min at 25°C. Images were captured under a microscope with a digital camera. For quantification of Hoechst 33342 staining, the percentage of Hoechst-positive nuclei per optical field (at least 50 fields) was calculated. Experiments were performed in triplicate.

### *In vivo* tumor growth assay

6-week-old female BALB/c nude mice were purchased from Henan Experimental Animals Centre Zhengzhou, China. EC9706 and KYSE30 cells were stably transfected with luciferase. The mice were randomly divided into three groups for each cell line, and each group consisted of five mice. Group si-lnc1: cells were transfected with lncRNATP73-AS1 siRNA1; Group NC: cells were transfected with nonsense siRNA; Group Blank: un-transfected cells. EC9706 and KYSE30 cells transfected with lncRNA TP73-AS1 siRNA1 were injected subcutaneously at 5×10^7^ cells. At 7, 14, 21, or 28 days, mice were injected intraperitoneally with D-luciferin (150 mg/kg) and analyzed using the Xenogen-IVIS Imaging System. The luciferase area of the xenograft tumor was defined as the region of interest (ROI), and the total signal in the ROI was quantified using the software, Living Image 3D (Xenogen). The Zhengzhou University Animal Care and Use Committee approved these protocols.

### Statistical analyses

All statistical analyses were performed using the SPSS 17.0 software. A student's *t*-test or one-way ANOVA was conducted for normally distributed data. Pearson Χ^2^ test was used to determine the correlation between lncRNA TP73-AS1 and BDH2 expression and the clinicopathologic features of patients. All data were expressed as the mean ± SD. Statistical significance was set at *P*<0.05.

## SUPPLEMENTARY TABLE


