# Supplementary material for: Knockdown of long non-coding RNA TP73-AS1 inhibits cell proliferation and induces apoptosis in esophageal squamous cell carcinoma
Source: Oncotarget. 2016 Jan 21;7(15):19960–74. doi: 10.18632/oncotarget.6963 (PMC4991431; doi:10.18632/oncotarget.6963)
Supplement: Supplementary file 1 [file oncotarget-07-19960-s001.pdf]

SUPPLEMENTARY TABLE

Supplementary Table S1: The sequences of siRNA

siRNA	positive-sense strand	antisense strand
LnRNATP73-AS1 siRNA1	5′GATCGCGTTCTGTGTGGAACTT ACTGGATCAAGAGTCCAGTAAGT TCCACACAGAATTTTTTCCAAA 3′	5′AGCTTTTGGAAAAAATTCTGTGTGGAA CTTACTGGACTCTTGATCCAGTAAGTTC CACACAGAACGC 3′
LnRNATP73-AS1 siRNA2	5′GATCGCGTAGACAGAGGTCAT CAGCCAGTCAAGAGCTGGCTGAT GACCTCTGTCTATTTTTTCCAAA 3′	5′AGCTTTTGGAAAAAATAGACAGAGGT CATCAGCCAGCTCTTGACTGGCTGATGA CCTCTGTCTACGC 3′
BDH2 siRNA1	5′GATCGCGGTGCCAAAGTCATA GCCATCAAGAGTGGCTATGAC TTTGGCACCTTTTTTCCAAA 3′	5′AGCTTTTGGAAAAAAGGTGCCAAAGT CATAGCCACTCTTGATGGCTATGACTT TGGCACCGC 3′
BDH2 siRNA1	5′GATCGCGCCAAGGCAGCCGT GATTGGTCAAGAGCCAATCAC GGCTGCCTTGGTTTTTTCCAAA 3′	5′AGCTTTTGGAAAAAACCAAGGCAGCC GTGATTGGCTCTTGACCAATCACGGC TGCCTTGGCGC 3′
